# Characteristics of Scar Formation After Intracerebral Hemorrhage in Aged Rats: Effects of Deferoxamine

**DOI:** 10.3390/cells14151127

**Published:** 2025-07-22

**Authors:** Xiongjie Fu, Yingfeng Wan, Ya Hua, Guohua Xi, Richard F. Keep

**Affiliations:** Department of Neurosurgery, University of Michigan, Ann Arbor, MI 48109, USA

**Keywords:** intracerebral hemorrhage, scar formation, deferoxamine, hemosiderin, astrocytes

## Abstract

Intracerebral hemorrhage (ICH), a severe stroke subtype common in the elderly, often results in high morbidity and mortality, with limited treatment options for long-term recovery. While glial scar formation is increasingly recognized as key to central nervous system (CNS) repair, its role and characteristics in the aging brain post-ICH remain unclear. This study investigated glial scar formation after ICH (100 μL autologous blood injected into the right basal ganglia model) in aged Fischer 344 rats and assessed the effects of deferoxamine (DFX) treatment. Histological and immunohistochemical analyses were conducted on days 7, 28, and 60 post-ICH using cell-specific and iron-related markers, with DFX administered at 100 mg/kg daily for 14 days in separate groups. Over time, the lesion core showed increased hemosiderin accumulation and astrogliosis. By day 60, the area of astrogliosis corresponded to an area with persistent neuronal loss (DARPP-32-negative). Glial composition shifted from microglia dominance on day 28 to astrocyte predominance by day 60. DFX treatment reduced iron deposition, astrogliosis, and DARPP-32-negative regions while enhancing oligodendrocyte presence. Iron-related markers (HO-1, ferritin, Perls’ staining) and PDGFRβ-positive fibrotic cells were concentrated in the scar core. These findings provide novel insights into scar formation after ICH in aged rats and suggest DFX as a potential therapy to improve outcomes in elderly stroke patients.

## 1. Introduction

Intracerebral hemorrhage (ICH) is a common subtype of stroke, with a higher incidence in the elderly population. It is often associated with substantial disability and elevated mortality rates [1]. While there is some recent evidence for a benefit with clot evacuation [2], therapeutic options remain limited [3]. Patients who survive ICH frequently experience persistent neurological impairments that significantly diminish their quality of life [4]. Therefore, promoting long-term neurological recovery has emerged as a critical therapeutic goal for ICH. Considerable attention has focused on the role of inflammation in ICH-induced acute/chronic injury and repair [5]. One such response to CNS injury is the proliferation and migration of reactive astrocytes to form a scar that surrounds damaged and inflamed tissue [6]. Such scar formation may form a barrier to axonal regeneration [7], but some studies have highlighted the essential role of scar formation in long-term tissue repair following central nervous system (CNS) injury [8,9,10,11]. In ICH, the specific characteristics of scar formation remain poorly understood, particularly in the aging brain.

After CNS injury, damage-associated factors initiate the development of a complex, multicellular, and compartmentalized scar structure [12]. Although such scarring may serve protective functions in the acute phase—such as limiting the spread of neuroinflammation [13]—it becomes a major barrier to axonal regeneration and functional recovery in the chronic phase [7]. Understanding the nature of scar formation after ICH may offer new avenues for therapeutic intervention aimed at improving long-term neurological outcomes.

Scar formation is influenced by a variety of post-injury factors [14]. Iron, a major byproduct of hematoma degradation, plays a central role in mediating secondary brain injury following ICH [15]. Our previous research demonstrated that deferoxamine (DFX), an iron chelator, can mitigate ICH-induced brain injury [16]. However, it remains unclear whether iron contributes to scar formation and whether DFX treatment can attenuate this process. Therefore, in this study, we used an aged rat model to address two primary objectives: (1) to investigate the cellular composition and characteristics of scar formation following ICH and (2) to determine whether DFX treatment can modulate scar formation in the chronic phase post-ICH.

## 2. Materials and Methods

### 2.1. Animal and ICH Model

All animal procedures in this study were approved by the University of Michigan Committee on Use and Care of Animals and complied with the United States Public Health Service’s Policy on Humane Care and Use of Laboratory Animals. Also, the study followed the ARRIVE guidelines for reporting in vivo animal experiments [17]. In total, 34 male Fischer 344 rats (NIH, Bethesda, MD, USA) aged 18 months were used in this study.

The ICH model was performed as previously described [18]. In brief, rats were anesthetized with pentobarbital (50 mg/kg; intraperitoneal), and a heating pad was used to maintain body temperature at 37.5 °C. Autologous blood was obtained from the right femoral artery. Rats were then transferred to a stereotaxic frame (Kopf Instruments; Tujunga, CA, USA). A 1 mm burr hole diameter was drilled in the skull, and 100 μL autologous blood was injected into the right basal ganglia using a 26-gauge needle (coordinates 0.2 mm anterior, 5.5 mm ventral, 3.5 mm lateral to bregma) at a rate of 10 μL/min. No anticoagulant was used before or during the injection procedure. After injection, the needle was kept in place for 10 min to prevent reflux. The burr hole was then sealed by bone wax, and the incision was sutured. This is a well-established and widely accepted experimental model that closely mimics key aspects of human spontaneous ICH, especially hypertensive hemorrhage, which frequently involves the basal ganglia. This blood injection ICH model reliably reproduces key pathophysiological features such as secondary inflammation, blood–brain barrier disruption, edema, and neurological deficits [19].

### 2.2. Experimental Groups

This study was conducted in two parts. In the first part, rats were euthanized on days 7, 28, and 60 after ICH (*n* = 6 each time point). In the second part, rats were randomly divided into ICH + vehicle and ICH + DFX groups (*n* = 8 each group). Rats were euthanized on day 60 after ICH. The ICH + DFX group was treated with DFX (100 mg/kg; intramuscular) every day for 14 days. The control ICH + vehicle group rats were treated with the same volume of saline. In all experiments, rat brains were obtained for histological analysis. Randomization was implemented with even and odd numbers.

### 2.3. Hematoxylin and Eosin Staining and Perls’ Prussian Blue Staining

Rats were humanely euthanized via intraperitoneal injection of pentobarbital at a dose of 100 mg/kg, followed by transcardial perfusion using 4% paraformaldehyde (PFA) chilled on ice. Subsequently, the brains were collected and fixed in 4% PFA for 24 h and then transferred to a 30% sucrose solution at 4 °C until fully equilibrated. The tissues were embedded in optimal cutting temperature (OCT) compound and cryosectioned into 18 μm coronal slices. Hematoxylin and Eosin (H&E) staining was performed following a previously established protocol [20].

For Perls’ Prussian blue staining, brain slides were incubated with iron stain solution (1:1 mixture of potassium ferrocyanide solution and 2% hydrochloric acid solution) for 3 min, followed by rinsing in distilled water. Subsequently, the brain slides were incubated with the Metal Enhanced DAB Substrate Kit (Thermo Fisher, Rockford, IL, USA, #34065) for 60 min. Finally, the slices were dehydrated, defatted, permeabilized, and fixed using alcohol and xylene at different concentrations.

### 2.4. Immunofluorescence

After drying, brain sections were treated with 0.3% Triton X-100 for 15 min to facilitate permeabilization. They were then blocked with 15% donkey serum for 1 h at room temperature. Following PBS rinses, the sections were incubated overnight at 4 °C with the respective primary antibodies: rabbit monoclonal anti-dopamine and cyclic AMP-regulated phosphoprotein, Mr 32 kDa (DARPP-32, Cell Signaling Technology, Danvers, MA, USA, #2306, 1:200), mouse monoclonal anti-Glial Fibrillary Acidic Protein (GFAP, Sigma, Saint Louis, MO, USA, MAB360, 1:500), goat polyclonal anti-Ionized Calcium-Binding Adapter Molecule 1 (Iba-1, Abcam, Boston, MA, USA, ab5076, 1:500), goat polyclonal anti-Oligodendrocyte Transcription Factor 2 (Olig2, R&D Systems, Minneapolis, MN, USA, AF2416, 1:400), and rabbit monoclonal anti-Platelet-Derived Growth Factor Receptor Beta (PDGFRβ, Abcam, Boston, MA, USA, ab32570, 1:400). After washing with PBS, sections were incubated with secondary antibodies: donkey anti-rabbit IgG, Alexa Flour 448 (Invitrogen, Carlsbad, CA, USA, A-21206, 1:500), donkey anti-mouse IgG, Alexa Flour 594 (Invitrogen, Carlsbad, CA, USA, A-21203, 1:500), and donkey anti-goat IgG, Alexa Flour 594 (Invitrogen, Carlsbad, CA, USA, A-11058, 1:500) at room temperature for 2 h. Finally, these sections were stained with DAPI (Sigma, Saint Louis, MO, USA, F6057) and observed under a fluorescence microscope.

### 2.5. Immunohistochemistry

Once the brain sections were air-dried, they were treated with 0.3% Triton X-100 for 15 min to permeabilize the tissue. This was followed by a 1 h incubation at room temperature with 10% goat serum to block nonspecific binding. Brain slices were incubated overnight with primary antibodies: rabbit monoclonal anti-DARPP-32 (Cell Signaling Technology, Danvers, MA, USA, #2306, 1:200), rabbit polyclonal anti-HO-1 (Enzo, Farmingdale, NY, USA, SPA-895-F, 1:500), and rabbit monoclonal anti-ferritin (Abcam, Boston, MA, USA, ab75973, 1:400) at 4 °C. After washing with methanol and H_2_O_2_, sections were incubated with the secondary antibodies: goat anti-rabbit IgG and Biotin (Invitrogen, Carlsbad, CA, USA, #31820, 1:500) at room temperature for 90 min. Sections were then washed with PBS and incubated with the tertiary antibody (ABC Peroxidase staining Kit, 1:100, #32020, Thermo Fisher Scientific, Rockford, IL, USA) at room temperature for 90 min. After rinsing in PBS, the sections were developed using stable DAB solution (Invitrogen, Carlsbad, CA, USA, #750118). The final steps involved graded dehydration, clearing in xylene, and fixation using a series of alcohol and xylene solutions of varying concentrations.

### 2.6. Cell Counting

Cell counting was performed by an investigator blinded to the experimental groups. For each rat, three brain sections were analyzed, and within each section, three distinct regions surrounding the hematoma were assessed. The average number of target cells was quantified using ImageJ software (National Institutes of Health, Bethesda, MD, USA, version 1.5).

### 2.7. Statistical Analysis

Data are expressed as mean ± standard deviation (SD). The Shapiro–Wilk test was employed to evaluate the normality of the datasets. For data following a normal distribution, unpaired Student’s t-tests were conducted; otherwise, the Mann–Whitney U test was used for non-parametric comparisons. Statistical significance was defined as a *p*-value of less than 0.05. All statistical analyses were carried out using GraphPad Prism version 8.0 (GraphPad Software Inc., San Diego, CA, USA).

## 3. Results

### 3.1. Evolution of Tissue Injury and Scar Formation from Day 7 to Day 60 After ICH

**Figure 1:**
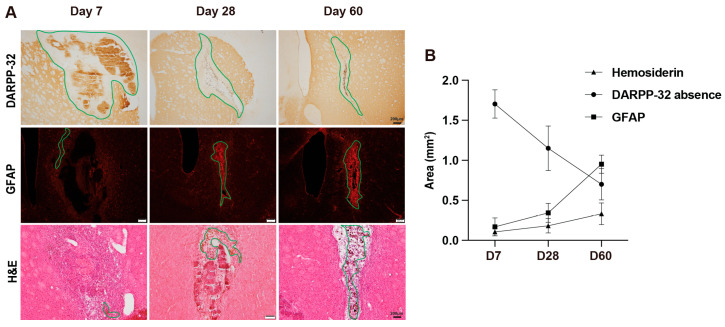
Histological overview of brain injury and scar formation in aged rats at multiple time points following ICH. (**A**) Representative images of DARPP-32 immunostaining, GFAP immunostaining, and H&E staining on days 7, 28, and 60 post-ICH. Green solid lines indicate the regions of interest used for quantification. In the first row, green solid lines outline regions lacking DARPP-32 expression. In the second row, they delineate areas of astrogliosis. In the third row, the green solid lines indicate regions of hemosiderin deposition. Scale bar = 200 μm. (**B**) Quantification of hemosiderin deposition, DARPP-32 absence area, and GFAP-positive staining area on days 7, 28, and 60 after ICH.

To investigate tissue injury and scar formation, DARPP-32, GFAP, and H&E staining were performed on brain sections on days 7, 28, and 60 post-ICH. ICH causes peri-hematomal injury, including the loss of medium-sized spiny neurons in the striatum that can be followed by the loss of DARPP-32 staining (Figure 1A). The size of the DARPP-32-negative region reduces over time (Figure 1B), with a glial (GFAP-rich) scar gradually forming in the center of the lesion (Figure 1A,B). By day 60, the area of the GFAP-rich scar and the area of DARPP-32-negative tissue are similar (Figure 1B). On H&E staining, there is an accumulation of brown hemosiderin particles and dense cellular nuclei in the central region of the lesion with time, indicating ongoing tissue damage and inflammatory response (Figure 1A). Hemosiderin deposition progressively increased from day 7 to day 60 post-ICH, reflecting continued degradation of blood products within the lesion core (Figure 1B). The hemosiderin-rich area of the lesion is smaller than the overall GFAP-rich scar.

**Figure 2:**
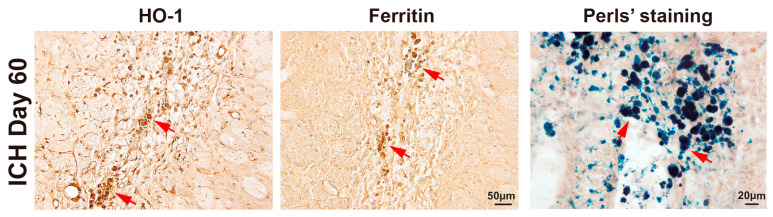
Representative immunohistochemical staining for HO-1 and ferritin on day 60 following ICH showing many positive cells within the scar. Red arrows indicate positive cells. Similarly, Perls’ staining reveals iron deposition within the core scar region. Red arrows indicate iron deposition. Scale bars = 20 µm or 50 µm as indicated.

After ICH, heme from the clot is degraded to biliverdin, CO, and iron by heme oxygenase 1 (HO-1). The iron released may be bound to ferritin, and the partial degradation of such ferritin results in hemosiderin. We observed a substantial accumulation of HO-1- and ferritin-positive cells within the scar region on day 60 post-ICH (Figure 2). To further characterize the brown hemosiderin deposits, Perls’ Prussian blue staining was performed, confirming their identity as iron-positive structures (Figure 2).

### 3.2. DFX Treatment Significantly Reduces Hemosiderin Deposition, Astrogliosis, and DARPP-32 Absence Area on Day 60 Post-ICH

**Figure 3:**
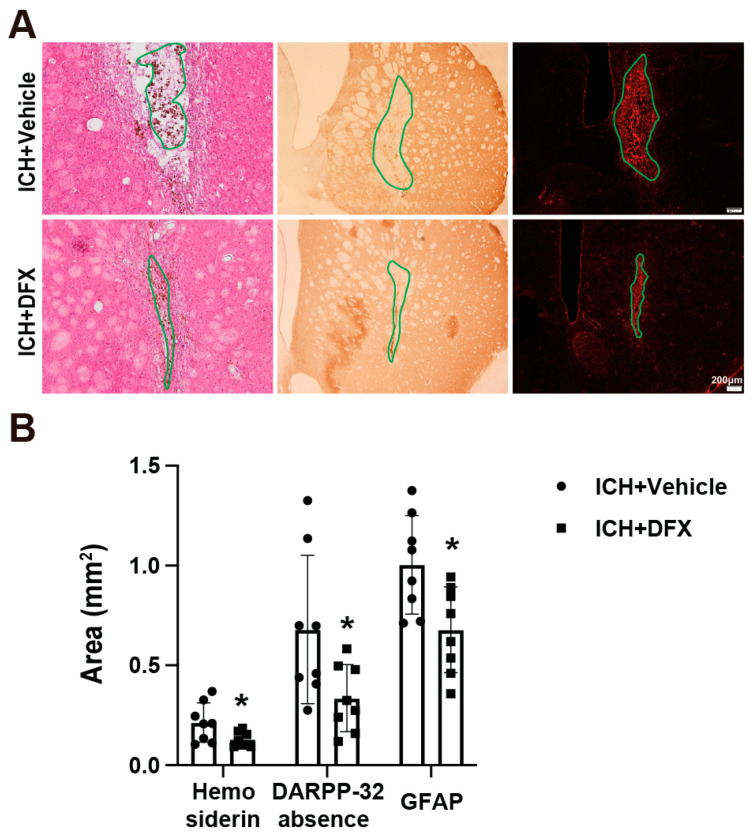
Effects of DFX on hemosiderin deposition and scar formation on day 60 after ICH in aged rats. (**A**) Representative images of H&E staining, DARPP-32 immunostaining, and GFAP immunostaining in ICH + vehicle and ICH + DFX (100 mg/kg) treatment groups on day 60 post-ICH. Green solid lines indicate regions used for quantification. In the first column, green solid lines indicate regions of hemosiderin deposition. In the second column, they outline regions lacking DARPP-32 expression. In the third column, the green solid lines delineate areas of astrogliosis. Scale bar = 200 μm. (**B**) Quantification of hemosiderin deposition, DARPP-32-absence area, and GFAP-positive area. *n* = 8 per group. Data are expressed as the mean ± SD. * *p* < 0.05 vs. ICH + vehicle groups by Student’s *t*-test.

Deferoxamine (DFX) treatment significantly reduced hemosiderin deposition in the lesion area on day 60 following ICH in aged rats (Figure 3A,B). Using DARPP-32 as a marker of structural integrity, we observed that DFX treatment decreased the DARPP-32-negative region, indicating attenuation of tissue damage (Figure 3A,B). Additionally, DFX treatment led to a significant reduction in the GFAP-positive area, reflecting reduced astrogliosis in the perihematomal region on day 60 (Figure 3A,B). These results suggest that DFX treatment effectively diminishes scar formation after ICH in aged rats.

### 3.3. Cellular Composition of the Scar in Aged Rats After ICH

**Figure 4:**
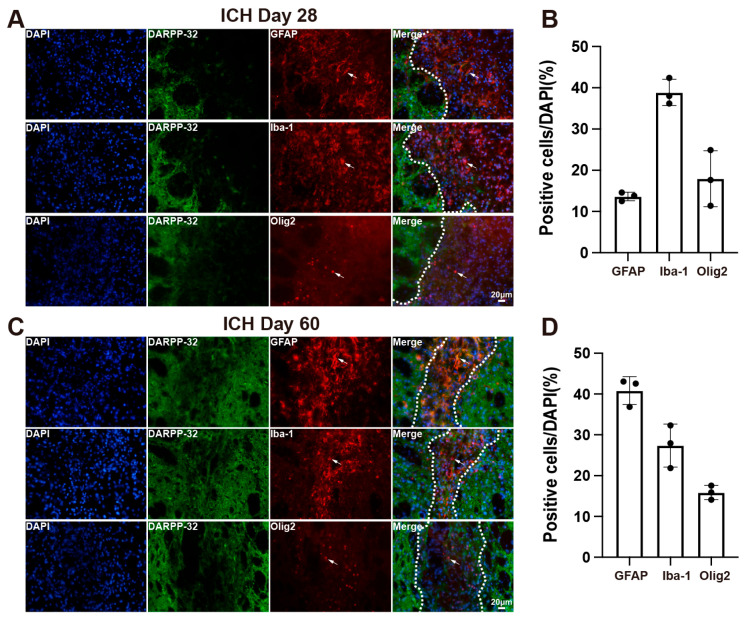
Glial cellular composition of the scar in aged rats on days 28 and 60 following ICH. (**A**) Representative immunofluorescence images of DARPP-32, GFAP, Iba-1, and Olig2 on day 28 post-ICH. White dashed lines delineate the scar border. White arrows indicate GFAP, Iba-1, and Olig2-positive cells. (**B**) Quantification of glial cell types within the scar region on day 28 (*n* = 3). (**C**) Representative immunofluorescence images of DARPP-32, GFAP, Iba-1, and Olig2 on day 60 post-ICH. White dashed lines delineate the scar border. White arrows indicate GFAP, Iba-1, and Olig2-positive cells. (**D**) Quantification of glial cell types within the scar region on day 60 (*n* = 3).

Using DARPP-32 as a structural marker to delineate the scar region, we performed immunofluorescence staining with multiple glial markers to characterize the cellular composition of the scar tissue in aged rats following ICH. The glial scar was composed of astrocytes, microglia, and oligodendrocytes. On day 28 post-ICH, Iba-1-positive microglia represented the predominant glial cell type within the scar region (Figure 4A,B). By day 60 post-ICH, however, the dominant glial component had shifted to GFAP-positive astrocytes (Figure 4C,D), indicating a temporal transition in the cellular makeup of the scar.

**Figure 5:**
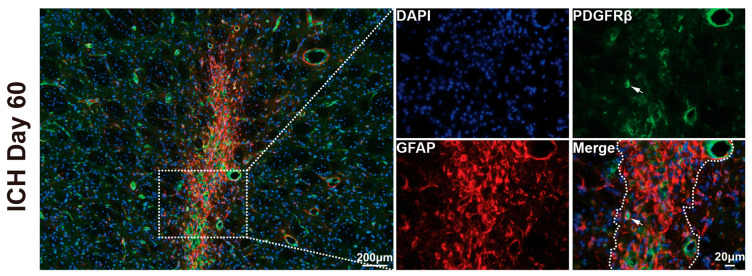
Representative immunohistochemical staining for PDGFRβ on day 60 following ICH. PDGFRβ is a marker for fibroblasts and pericytes. Fibrotic components were predominantly localized in the scar core. White dashed lines and boxes indicate regions selected for higher magnification. In high-magnification images, dashed lines delineate the scar boundary. White arrows indicate PDGFRβ-positive cells. Scale bars = 200 µm or 20 µm as indicated.

Fibrotic components also represent a major component of the mature scar. To examine this, we used PDGFRβ, a marker for fibroblasts and pericytes. Notably, PDGFRβ-positive fibrotic tissue was primarily located in the core of the scar on day 60 post-ICH, indicating fibroblast involvement in the chronic scarring process (Figure 5).

### 3.4. DFX Treatment Modulates Glial Composition Within the Scar Following ICH in Aged Rats

**Figure 6:**
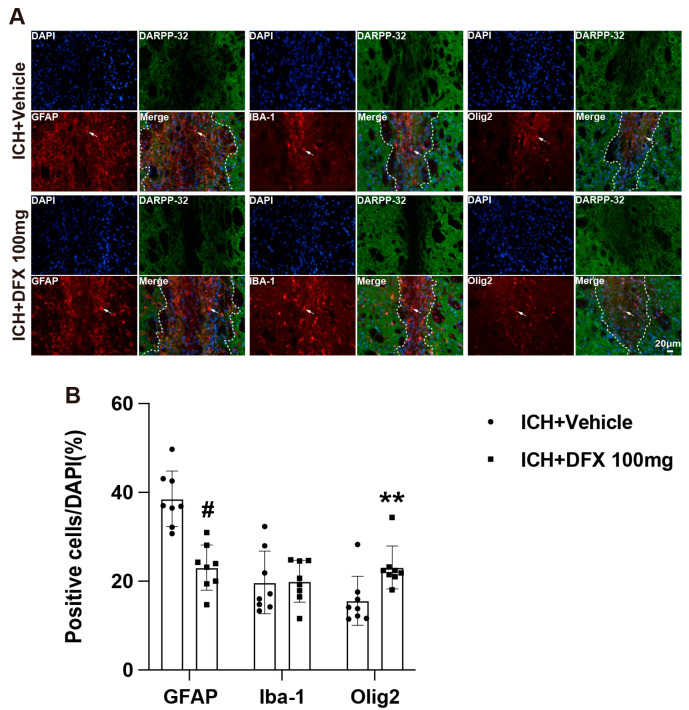
DFX alters glial cellular composition within the scar (DARPP-32-negative area) on day 60 following ICH. (**A**) Representative immunofluorescence images of DARPP-32, GFAP, Iba-1, and Olig2 in aged ICH rats treated with vehicle or DFX (100 mg) on day 60. White dashed lines delineate the scar boundary. White arrows indicate GFAP, Iba-1, and Olig2-positive cells. (**B**) Quantification of glial cell types (as a % of DAPI cells) within the scar. *n* = 8 per group. Data are presented as mean ± SD. # *p* < 0.01 vs. ICH + vehicle group by Student’s *t*-test and ** *p* < 0.01 vs. ICH + vehicle group by the Mann–Whitney U test.

To investigate the effect of iron chelation on scar composition in aged rats, we administered DFX. DFX treatment significantly reduced the proportion of GFAP-positive astrocytes within the scar region and increased the number of Olig2-positive oligodendrocytes on day 60 post-ICH. These changes suggest that DFX may promote a more regenerative glial environment conducive to neurofunctional recovery. DFX treatment did not significantly alter the proportion of Iba-1-positive microglia (Figure 6A,B).

## 4. Discussion

This study provides novel insights into scar formation after ICH in aged rats and evaluates the effects of DFX. Our main findings are as follows: (1) hemosiderin deposition and astrogliosis within the scar progressively increased from day 7 to day 60 post-ICH, while the DARPP-32-negative (staining absence) area gradually decreased; (2) DFX treatment reduced hemosiderin deposition, astrogliosis, and the GFAP-negative area; (3) astrocytes constituted the primary glial component of the scar, accompanied by scattered microglia, oligodendrocytes, and fibrotic elements; and (4) DFX was found to reduce astrocyte presence and increase oligodendrocyte content within the scar region.

Following CNS injury, a specialized structure known as the glial scar forms within the lesion area, driven by complex pathophysiological processes [21,22]. The scar resulting from injury comprises intricate cellular components and structures, including glial and fibrotic elements [23]. The role of scar formation after CNS injury remains controversial. While some studies suggest that the glial scar acts as a physical and molecular barrier to axonal regrowth, other evidence highlights its potential benefits in supporting tissue repair and promoting axon regeneration under certain conditions [8,9,24]. ICH, a significant CNS injury disease, also induces glial scar formation. Although several studies have investigated such scar formation, most of this research has focused on young animal models [25,26]. As a result, the specific characteristics and cellular dynamics of scar formation after ICH in aged animals remain poorly understood.

In this study, we investigated the progression of scar formation following ICH in aged rats at multiple time points. Notably, we observed the accumulation of numerous brown particles, identified as hemosiderin, within the central lesion area, particularly during the chronic phase of ICH. As the hematoma resolves, various metabolic byproducts are released, with hemosiderin being a prominent ferritin-derived end-product frequently observed during the late stages of ICH recovery [27,28]. On day 60 post-ICH, we identified a distinct DARPP-32-negative region in the ipsilateral basal ganglia, indicating structural damage. This DARPP-32-negative area exhibited a more defined boundary compared to the region of astrogliosis. Moreover, from day 7 to day 60 post-ICH, we found a progressive increase in both hemosiderin deposition and astrogliosis within the scar region, accompanied by a gradual reduction in the DARPP-32-negative area, suggesting partial tissue remodeling over time.

DFX, an iron chelator, has been shown to exert neuroprotective effects and is considered a promising therapeutic agent in various ICH animal models, including young adult rats, aged rats, and piglets [29]. DFX treatment has been demonstrated to reduce brain edema, neuronal death, and iron accumulation following ICH [30,31,32,33]. As previously noted, hemosiderin—an end-product of hematoma degradation—accumulates in the lesion core. Although this study did not fully elucidate the specific role of iron and hemosiderin in scar formation, our findings indicate that iron chelation with DFX treatment significantly reduces hemosiderin deposition. Scar area is another critical factor influencing long-term neurofunctional recovery after ICH. Our previous work has shown that DFX can reduce lesion cavity size post-ICH [34]. Consistent with these findings, the present study used the DARPP-32-negative/structurally damaged area as a surrogate for scar tissue and revealed that DFX treatment significantly decreased scar size. This conclusion is further supported by the observation that DFX also reduced the GFAP-positive area, indicating attenuation of reactive astrogliosis.

Previous studies have suggested that astrocytes are a major component of the scar formed following CNS injury [35], and astrogliosis is often used to demarcate the scarred region. However, using astrogliosis alone to define the boundaries of the scar is neither entirely reliable nor precise, as astrocytes are also abundantly present in normal brain tissue. In our prior research, we demonstrated that DARPP-32 serves as a reliable marker for basal ganglia neurons. This marker allows for accurate quantification of neuronal loss induced by ICH during both the acute and subacute phases [36]. DARPP-32 is a well-established marker of striatal projection neurons, which comprise over 95% of striatal neurons and include both D1 dopamine receptor-expressing direct pathway neurons and D2 receptor-expressing indirect pathway neurons [37]. While DARPP-32 is a reliable marker for these projection neurons, it is not expressed in intrinsic striatal interneurons, such as cholinergic or parvalbumin-positive GABAergic interneurons [38], which limits its ability to reflect changes in these populations. Previous studies have shown that D2-expressing striatopallidal neurons tend to be more susceptible to neurodegeneration than D1-expressing striatonigral neurons in various models, potentially due to lower calcium-buffering capacity, distinct receptor expression, and higher sensitivity to glutamatergic excitotoxicity [39,40]. In ICH specifically, iron toxicity, complement activation, and reactive oxygen species accumulation may further amplify this vulnerability, particularly in neurons with high metabolic demand or impaired mitochondrial resilience. Conversely, intrinsic cholinergic interneurons, which are DARPP-32-negative, appear to be relatively resistant to stroke-induced damage, possibly due to hyperpolarization-activated cation current [41].

Using DARPP-32 as a marker to delineate the extent of scar after ICH, we investigated the dynamic changes in scar formation composition over time. Notably, our findings differ from previous studies on post-ICH scar formation. On day 28 post-ICH, we observed that the predominant glial cells within the scar region were Iba-1-positive microglia. This discrepancy may be attributable to methodological differences, as earlier studies primarily utilized collagen injection ICH models in young animals [25,26]. By day 60 post-ICH, the dominant glial population had shifted to GFAP-positive astrocytes, which aligns with established findings from CNS injury models. Based on these observations, we suggest that day 60 represents an optimal time point for studying mature scar formation in aged rats following ICH. In addition to glial elements, fibrotic cells are also key constituents of the scar. In this study, we identified that PDGFRβ-positive fibrotic cells were primarily localized in the core of the scar on day 60 post-ICH. However, due to the dual expression of PDGFRβ in both fibroblasts and pericytes, we were unable to definitively determine the predominant fibrotic cell type present in the lesion core.

Iron is a significant factor in ICH-induced brain injury during the acute phase, as it is released into the brain tissue following the lysis of red blood cells within the hematoma [42]. However, its role in scar formation after ICH remains unclear. HO-1 is a key enzyme involved in the degradation of Hb, while ferritin functions as an intracellular iron-binding protein [43,44]. In this study, we observed substantial accumulation of HO-1- and ferritin-positive cells within the scar region. Moreover, hemosiderin—a byproduct of iron metabolism—was predominantly localized in the core of the scar. These findings suggest a strong association between iron metabolism and the process of scar formation following ICH.

In this study, we further investigated the involvement of iron in scar formation following ICH. Our results demonstrated that DFX treatment in aged rats significantly decreased the proportion of GFAP-positive astrocytes and increased the proportion of Olig2-positive oligodendrocytes within the scar region. The astrocytic barrier formed by GFAP-positive cells at the periphery of the scar is a well-recognized obstacle to axonal regeneration, whereas Olig2-positive oligodendrocytes are known to support axonal growth. While we did not directly label regenerating axons, the increased presence of Olig2+ cells within the scar region and decreased astrocytic scarring are suggestive of a more permissive environment for axonal growth, as supported by previous studies [45,46]. Thus, the modulatory effect of DFX on scar cellular composition may substantially contribute to long-term neuroprotection/increased repair in ICH. While the iDEF trial of DFX in human ICH did not find evidence of protection at 90 days, it did at 180 days [47].

Taken together, our results highlight the dynamic and complex nature of glial scar formation in aged rats after ICH and demonstrate the beneficial effects of DFX in modulating scar components. These findings may have translational relevance, particularly for aged human patients at high risk for poor recovery after hemorrhagic stroke. Future studies should further investigate the molecular mechanisms by which DFX influences scar cellular composition and determine whether these changes translate into long-term functional benefits.

## 5. Conclusions

In conclusion, glial scar formation after ICH in aged rats is a dynamic process involving iron deposition and reactive gliosis. DFX treatment modulates scar architecture by reducing astrocytic dominance and promoting oligodendrocyte presence, potentially creating a more favorable environment for neural repair. These findings offer novel insights into the pathophysiology of ICH in the aged brain and highlight DFX as a promising therapeutic agent for improving outcomes in elderly stroke patients.

## Data Availability

All supporting data are contained within the article. The datasets used and/or analyzed in this study are available from the corresponding author upon reasonable request.

## Abbreviations

The following abbreviations are used in this manuscript:

ICH	Intracerebral Hemorrhage
Hb	Hemoglobin
HO-1	Heme Oxygenase-1
Iba-1	Ionized Calcium-Binding Adapter Molecule 1
Olig2	Oligodendrocyte Transcription Factor 2
PDGFRβ	Platelet-Derived Growth Factor Receptor Beta
DARPP-32	Dopamine- and cAMP-Regulated Phosphoprotein of 32 kDa
CNS	Central Nervous System
DFX	Deferoxamine
PFA	Paraformaldehyde
OTC	Optimal Cutting Temperature
H&E	Hematoxylin and Eosin
NIH	National Institutes of Health
SD	Standard Deviation
DAB	Diaminobenzidine
DAPI	4′,6-Diamidino-2-Phenylindole
PBS	Phosphate-Buffered Saline
ARRIVE	Animal Research: Reporting of In Vivo Experiments
GFAP	Glial Fibrillary Acidic Protein
